# Rodents Inhabiting the Southeastern Mu Us Desert May Not Have Experienced Prolonged Heat Stress in Summer 2022

**DOI:** 10.3390/ani13132114

**Published:** 2023-06-26

**Authors:** Yang-Yang Guo, Shan-Shan Wang, Xinyue Wang, Wei Liu, Deli Xu

**Affiliations:** 1 College of Life Sciences, Qufu Normal University, Qufu 273165, China; shg2019@qfnu.edu.cn (Y.-Y.G.);; 2 Qufu Municipal Bureau of Agriculture and Rural Affairs, Qufu 273165, China; 3 State Key Laboratory of Integrated Management of Pest Insects and Rodents, Institute of Zoology, Chinese Academy of Sciences, Beijing 100101, China; 4 University of Chinese Academy of Sciences, Beijing 100049, China

**Keywords:** summer heat, behavioral adjustments, physiological adjustments, Midday gerbil (*Meriones meridianus*), Mongolian gerbil (*Meriones unguiculatus*)

## Abstract

**Simple Summary:**

Rodents in the Mu Us Desert may respond to increasing summer heat through behavioral or physiological adjustments. We studied the ambient temperatures that *Meriones meridianus* and *Meriones unguiculatus* might experience in their habitats. The results showed that both the heat-sensitive *Meriones meridianus* and the heat-tolerant *Meriones unguiculatus* were threatened by extreme summer heat. In addition, although vegetation and rodent burrows increased the variety of ambient temperature in the habitat, only rodent burrows were effective in providing protection from the stress of extreme summer heat. Therefore, behavioral adjustments may be the major way in which small rodents in this region avoid continuous heat stress in summer.

**Abstract:**

Climate change combined with human activities has altered the spatial and temporal patterns of summer extreme heat in the Mu Us Desert. To determine how those rodents living in the desert respond to increased extreme heat in summer, in July 2022, during the hottest month, we examined the rodent species, vegetation coverage, and small-scale heterogeneity in ambient temperature in the southeastern Mu Us Desert. The results showed that *Meriones meridianus*, *Meriones unguiculatus*, and *Cricetulus longicaudatus* were found in the study area, where the vegetation coverage is 33.5–40.8%. Moreover, the maximum temperature of the desert surface was 61.8 °C. The maximum air temperature at 5 cm above the desert surface was 41.3 °C. The maximum temperature in the burrow at a depth of 15 cm was 31 °C. *M. unguiculatus* might experience 4–9.3 h of heat stress in a day when exposed outside the burrow, whereas *M. meridianus* would experience 8.5–10.8 h of heat stress. Yet, inside the burrow, both species were barely exposed to heat stress. In conclusion, adjustments in behavioral patterns can be the main way that rodents in the Mu Us Desert adapt to the extreme heat in the summer.

## 1. Introduction

Hot summer is becoming a deadly season for many mammals on Earth [1,2]. Many regions of the world experienced record-breaking heat events in 2022 [3,4,5]. Moreover, the frequency and severity of extreme heat events are predicted to increase in the future due to global climate change [6]. It is necessary to understand how mammals in the wild will respond to increasing extreme summer heat.

Generally, facing extreme heat, small mammals will first reduce the stress on their thermoregulatory system through behavioral adjustments. For example, desert rodents tend to search shaded cooler places by moving around flexibly during the hottest part of the day [7]. Furthermore, some thermoregulating behaviors, such as specific postures, panting, and saliva spreading, contribute to the body heat dissipation [8]. In addition, the mammals also cope with extreme heat through physiological adjustments. Some studies indicated that adjustments in peripheral thermoeffectors and the thermoregulatory center allow animals to maintain a stable body temperature by altering the rate of heat production and heat loss [9,10,11]. Moreover, the degree of physiological tolerance to heat has become an important factor affecting the survival of desert rodents, especially when behavior does not effectively regulate the body heat balance.

The Mu Us Desert is located at the northern margin of the Loess Plateau in northwest China, covering an area of about 40,000 km^2^ [12,13]. The desert is mainly influenced by arid and semi-arid climates, which is a typical fragile ecological zone and very sensitive to climate change [14,15]. On the one hand, climate change is usually considered to be an important driving force of habitat change for desert rodents. For example, increasing extreme heat may have severe impacts on the thermal environment experience of animals [16]. On the other hand, human activities, such as grazing and reclamation, have aggravated the desertification in the 1970s and 1980s [17]. However, with the implementation of restoration policies approved since the 1990s, the transformation of desertification has improved [18,19]. Obviously, multiple and interacting factors create a diverse habitat for rodents in the Mu Us Desert, in which rodents should evolve all kinds of behavioral and/or physiological ways to adjust their heat adaption.

The Mu Us Desert is a common habitat for multiple rodent species, including the Mongolian gerbil (*Meriones unguiculates*), Midday gerbil (*Meriones meridiaus*), and Long-tailed hamster (*Cricetulus longicaudatus*) [20]. *M. unguiculatus* shows a high degree of tolerance for heat [21]. The range of thermal neutrality extends to 38.9 °C [22], which is perhaps one of the highest reported for small rodents in the literature. Mechanistically, this species can maintain body heat balance through the adjustment of the mitochondrial membrane, aquaporin expression in the kidney, or metabolic heat production when exposed to acute or chronic heat [22,23,24,25]. In addition, evidence from field observations suggests that *M. unguiculatus* is usually active during the day [26]. In contrast, *M. meridianus* has relatively low tolerance to heat, with a range of thermal neutrality extending to 30.1 °C [27]. This species is nocturnal [28]. As with *M. meridianus*, *C. longicaudatus* is nocturnal, but available data on its tolerance to heat are scarce [29,30]. In fact, the upper critical temperature of thermal neutrality for most of the small rodents in the Mu Us Desert is 30–38 °C [31,32,33,34]. In the present study, we are interested in whether or not the heat-tolerant *M. unguiculatus* adopted different coping strategy from the heat-sensitive *M. meridianus* in response to increased extreme summer heat. We found that behavioral adjustments could address the needs of *M. unguiculatus* and *M. meridianus* to adapt to extreme summer heat in 2022.

## 2. Materials and Methods

### 2.1. Study Area

**Figure 1:**
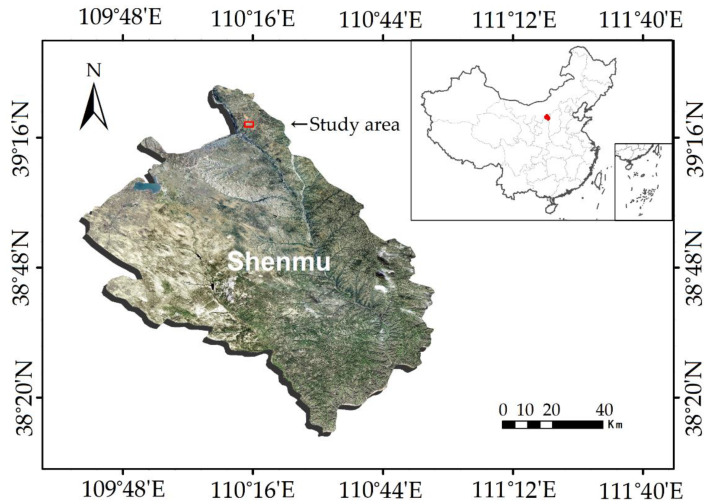
Location of the study area, Shenmu County, China. The red graph in the upper right of the figure, the location of Shenmu City.

Field experiments were conducted in the Daliuta forest conservancy area, which is located in Shenmu County, Shanxi Province, northwest China (Figure 1). The study area (39°18′ N, 110°41′ E, 1180–1224 m above sea level) lies in the southeast of the Mu Us Desert. This region is covered by sand dunes, sand sheets, and herbaceous and small shrub communities. The dominant vegetation type is shrub communities, such as *Salix cheilophila*, *Caragana intermedia*, and *Artemisia ordosica*. The climate there is generally characterized by extreme high temperature, strong sunlight, high evaporation, and erratic rainfall during the summer season [14]. The hottest month is July. All field work in this study was carried out in July 2022.

### 2.2. Vegetation Analysis

Vegetation surveys were conducted at three study sites. The size of the study site was 90 m × 60 m. A square of 5 m × 5 m was randomly sampled in the site to survey shrub communities. In addition, 3 squares of 1 m × 1 m across the square for herbs were sampled to investigate herbaceous plants [35]. The height and coverage of the plants were measured using a metric tap (to 1 mm). Plant species were identified by their morphologies.

### 2.3. Rodent Surveys

Rodents were captured using animal live traps [32]. In total, 50 wire-mesh traps (28 cm × 13 cm × 10 cm) were placed at each study site with 2–3 m spacing between traps. They were located close to rodent burrows or where there were animal tracks, such as rodent footprints or feces. For traps not under vegetation, a shade area was created with *Salix cheilophila* branches and leaves to protect the animals from excessive exposure to sunlight. All traps were set at 6 p.m. with peanuts as bait. The trap was triggered when the rodent ate the bait. All traps were checked on 2 consecutive days at 6 a.m., 12 a.m., and 6 p.m. All captured rodents were placed separately in a clear plastic box (43 cm × 34 cm × 30 cm) to record their body mass and morphology. The species of each rodent was identified based on characteristic morphological features. Finally, all rodents were released at the place where they were caught.

### 2.4. Determination and Analysis of Ambient Temperature

Near-surface ambient temperatures at the study sites were determined daily and at various heights over a period of two weeks. Ambient temperatures at 50 cm, 5 cm, and 0 cm above the ground (T_50cm_, T_5cm_, and T_0cm_) were measured using temperature loggers (RC-4, Elitech, Xuzhou, China). The ambient temperatures were recorded every 15 min. The data obtained were used for further analysis when the weather conditions were sunny and clear for 36 consecutive hours. The ambient temperature extremes were calculated by averaging 3 consecutive maximum or minimum ambient temperatures over a 24 h period.

At the same time, the desert surface temperature was measured with an infrared thermal imager (C200 Pro, InfiRay, Yantai, China) and temperature logger. The ratio of the desert surface temperature under vegetation (T_UV_) extremes to the desert surface temperature with no vegetation (T_NV_) extremes was used to measure the effect of vegetation cover on desert surface temperature. Moreover, correlations between T_5cm_, T_NV_, and T_UV_ were evaluated by simple linear regression analysis.

Ambient temperatures inside the burrow (T_burrow_) were measured using temperature loggers. The temperature probe was placed at a depth of 15 cm from the desert surface, 20 cm away from the opening of the rodent burrow. The depth of the temperature probe was determined by averaging the 10 rodent burrows surveyed in the field. In addition, to understand the ambient temperature characteristics of the rodent burrow, desert soil temperatures were measured with a digital thermometer (TES-1310, TES Electrical Electronic Corp., Taipei, China).

Linear regression analysis was performed to assess the linearity between air temperature (T_air_) and T_50cm_, T_5cm_, and T_0cm_, and T_UV_. T_air_ data were obtained from the official website (www.nmc.cn, accessed on 29 July 2022). The mean values of Shenmu and Ejin Horo Banner T_air_ were used in this study.

### 2.5. Determination of the Extreme Heat

Extreme heat was determined by the thermoneutral zone (TNZ) of the rodents [31]. The *M. unguiculatus* has a TNZ of 26 to 38 °C [24]. Therefore, the species was experiencing extreme heat when the ambient temperature was above 38 °C. In addition, the TNZ of the *M. meridianus* ranges from 25.5 to 30.1 °C [27]. This species was experiencing extreme heat when the ambient temperature was greater than 30.1 °C.

### 2.6. Statistical Analyses

All statistical analyses were performed using IBM SPSS.v.20.0 software. Plant heights, the difference between ambient temperature extremes and T_air_ extremes, the ratio of T_UV_ extremes/T_NV_ extremes, and the ratio of T_burrow_ extremes/T_0cm_ extremes were provided as mean ± SD. The general linear model was used to regress measures of ambient temperature against T_air_. Differences in other indicators between 2 groups were analyzed using the independent sample *t*-test. *p* values of less than 0.5 were considered statistically significant.

## 3. Results

### 3.1. Plant Species, Height, and Coverage

The plant species recorded at each site are shown in Table 1. In site 1, the vegetation coverage was 40.82%. *Salix cheilophila* was the major species, with a height of 1.904 ± 0.256 m and coverage of 40.44%. A small amount of *Artemisia ordosica* was found, with a height of 0.7 m and coverage of 0.38%. In site 2, the vegetation coverage was 33.53%. *Caragana intermedia* was the major species, with a height of 1.625 ± 0.178 m and coverage of 22.17%. *Artemisia ordosica* was the minor species, with a height of 0.535 ± 0.176 m and coverage of 11.36%. A small amount of *Cynanchum thesioides* was distributed in the interspace of shrubs. In site 3, the vegetation coverage was 36.67%. *Artemisia ordosica* was the minor species, with a height of 0.610 ± 0.188 and coverage of 26.40%. *Caragana intermedia* was the minor species, with a height of 1.165 ± 0.191 and coverage of 7.85%. *Amygdalus pedunculata* was another minor species, with a height of 0.700 ± 0.100 and coverage of 2.42%. There were also small amounts of *Cynanchum thesioides* and *Psammochloa villosa* mixed among the bushes.

### 3.2. Small Mammals Living in the Study Area

**Figure 2:**
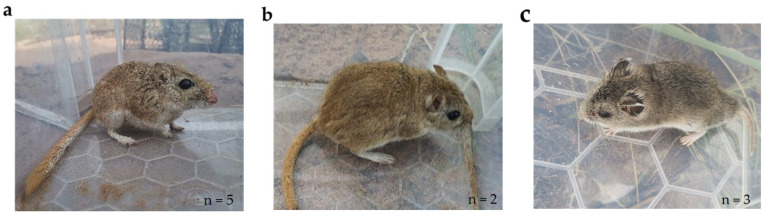
Rodents captured in the study area. (**a**) Midday gerbils; (**b**) Mongolian gerbils; (**c**) Long-tailed hamsters. All captured rodents were found during the 6 am checks.

Mongolian gerbil, Long-tailed hamster, and Midday gerbil were found in the study area (Figure 2). No animals were captured at site 1. Three Midday gerbils and two Long-tailed hamsters were captured at site 2. Two Mongolian gerbils, two Midday gerbils, and one Long-tailed hamster were captured at site 3. All rodents were captured before 6 a.m.

### 3.3. Ambient Temperature Variation in Near-Surface Space

**Figure 3:**
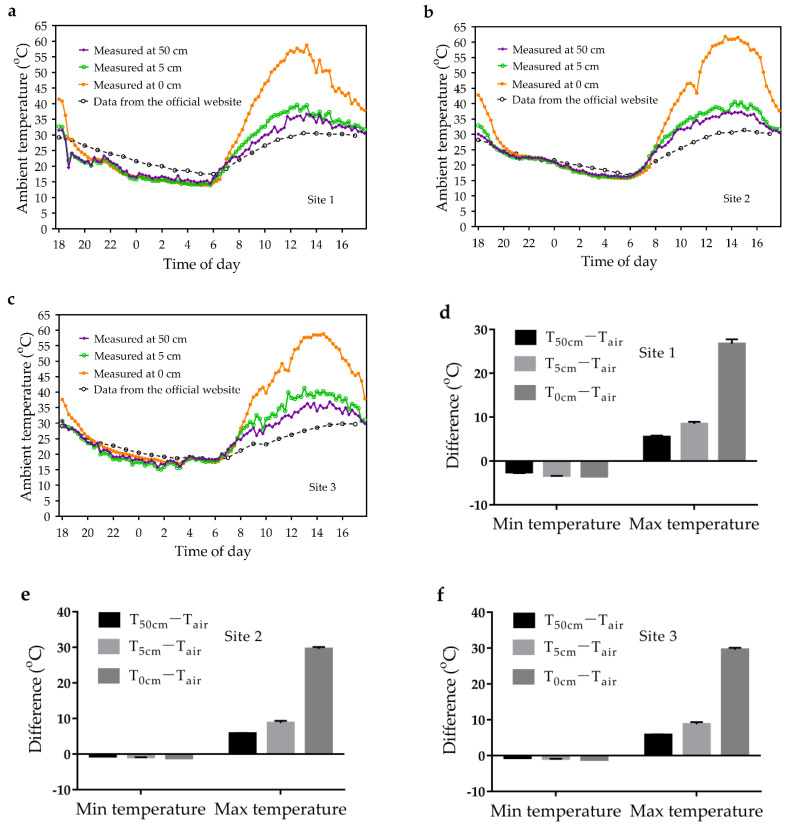
Near-surface ambient temperature variation. (**a**–**c**) Changes in near-surface ambient temperature at 3 study sites (measured on different dates) over 24 h; (**d**–**f**) The difference (mean ± SD) between ambient temperature extremes (*n* = 3) and T_air_ extremes. T_xcm_ (x = 50, 5, 0), ambient temperature measured at x cm above the ground; T_air_, air temperature from the official website.

The daily changes in near-surface ambient temperatures are shown in Figure 3a–c. The near-surface ambient temperatures decreased during the night. They started to increase after sunrise and reach a maximum between 1 and 3 pm. Compared to the T_air_ from the official website, the near-surface ambient temperatures were lower at night (Figure 3d–f). The difference between the lowest T_50cm_, T_5cm_, and T_0cm_ and the lowest T_air_ were −2.5 °C, −3.3 °C, and −3.5 °C, respectively (Figure 3d). In contrast, during the day, the near-surface temperatures were higher and showed a greater variation (Figure 3d–f). The difference between the highest T_50cm_, T_5cm_, and T_0cm_ and the highest T_air_ were 6.1 °C, 10.17 °C, and 29.6 °C, respectively (Figure 3e,f).

### 3.4. Effect of Vegetation on Desert Surface Temperature

**Figure 4:**
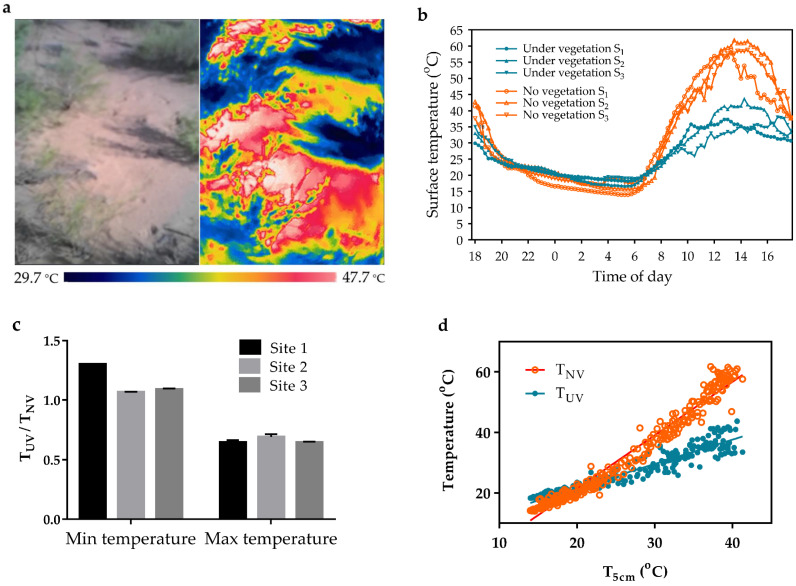
The influence of vegetation cover on desert surface temperature. (**a**) Desert surface temperature recorded by an infrared thermal imager. (**b**) Diurnal variation of desert surface temperature at three study sites. (**c**) Ratio (mean ± SD, *n* = 3) of T_UV_ extremes/T_NV_ extremes. (**d**) Linear regression between T_5cm_ and T_NV_ and T_UV_ (T_NV_, Y = 1.763x – 13.79, R^2^ = 0.9673; T_UV_, Y = 0.805x + 5.456, R^2^ = 0.9144). S_1–3_, site 1–3. T_NV_, desert surface temperature without vegetation; T_UV_, desert surface temperature under vegetation; T_5cm_, ambient temperature measured at 5 cm above the ground.

Vegetation cover had a regulatory function of desert surface temperature (Figure 4a,b). At night, the ratio of the lowest T_UV_ to the lowest T_NV_ was 1.07–1.30 (Figure 4c). During the day, the ratio of the highest T_UV_ to the highest T_NV_ was 0.65–0.69 (Figure 4c). Linear regression between T_5cm_ and T_NV_ and T_UV_ (T_NV_, Y = 1.763x – 13.79, R^2^ = 0.9673; T_UV_, Y = 0.805x + 5.456, R^2^ = 0.9144) were performed (Figure 4d). Vegetation cover regulated the desert surface temperature and brought it close to T_5cm_.

### 3.5. Linear Relationship between Tair and Near-Surface Ambient Temperature 

**Figure 5:**
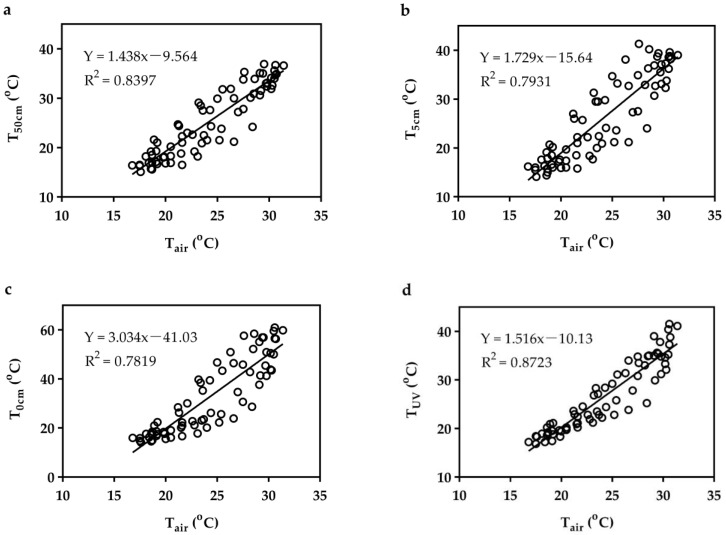
Linear regression between T_air_ and T_50cm_ (**a**), T_5cm_ (**b**), T_0cm_ (**c**), and T_UV_ (**d**). T_air_, air temperature from the official website; T_xcm_ (x = 50, 5, 0), ambient temperature measured at x cm above the ground; T_UV_, desert surface temperature under vegetation.

There was a positive linear relationship between T_air_ and T_50cm_ (Y = 1.438x – 9.564, R^2^ = 0.8397), T_5cm_ (Y = 1.729x – 15.64, R^2^ = 0.7931), T_0cm_ (Y = 3.034x – 41.03, R^2^ = 0.7819), and T_UV_ (Y = 1.516x – 10.13, R^2^ = 0.8723) (Figure 5). Near-surface ambient temperature could be predicted from the linear regression function with T_air_.

### 3.6. Ambient Temperature in Rodent Burrow 

**Figure 6:**
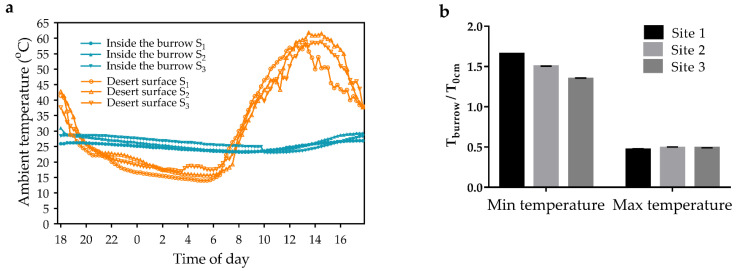

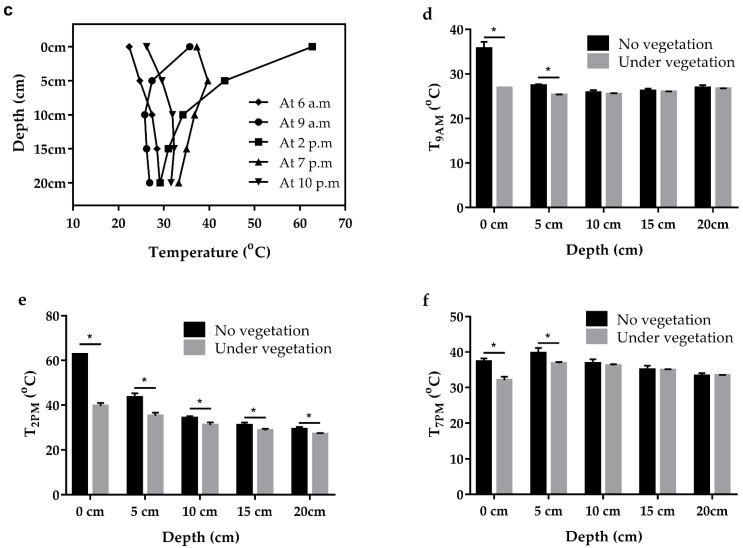
Rodent burrow regulation of ambient temperature. (**a**) Diurnal variation in ambient temperature on the desert surface and in rodent burrows. (**b**) Ratio (mean ± SD, *n* = 3) of T_burrow_ extremes/T_0cm_ extremes. (**c**) Variation of desert soil temperature with depth at different times of the day. (**d**–**f**) Effect of vegetation cover on desert soil temperature (mean ± SD, *n* = 3) during the day. S_1–3_, site 1–3. T_9am_, T_2pm_, and T_7pm_ desert soil temperature measured at 9 a.m., 2 p.m., and 7 p.m. T_burrow_, ambient temperature inside the burrow; T_0cm_, ambient temperature measured at 0 cm above the ground. Statistical significance is indicated by asterisks.

The daily changes in ambient temperature on the desert surface and in the rodent burrow are shown in Figure 6a. At night, the ratio of the lowest T_burrow_ to the lowest T_0cm_ was 1.35–1.66 (Figure 6b). During the day, the ratio of the highest T_burrow_ to the highest T_0cm_ was 0.47–0.49 (Figure 6b). Moreover, daily variations in desert soil temperature declined with increasing soil depth (Figure 6c). At 9 a.m. and 7 p.m., the effect of vegetation on the soil temperature was not significant at depths greater than 10 cm (Figure 6d,f). At 2 p.m., the difference in soil temperature due to vegetation cover was less than 2.3 °C at depths greater than 10 cm (Figure 6e).

### 3.7. Distribution of Extreme Heat 

**Figure 7:**
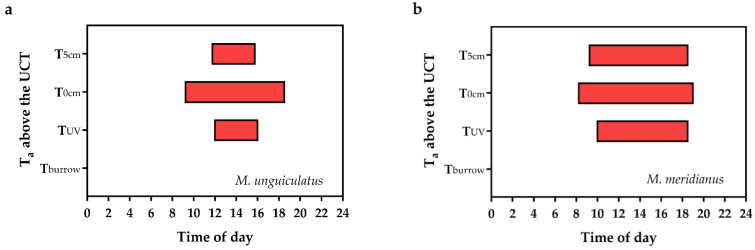
Distribution of extreme heat in time and space within a day. (**a**) Extreme heat determined by the upper critical temperature (UCT) of the TNZ in *M. unguiculatus*. (**b**) Extreme heat determined by the UCT of the TNZ in *M. meridianus*. T_0cm_ and T_5cm_ ambient temperature measured at 0 cm and 5 cm above the ground. T_UV_, desert surface temperature under vegetation; T_burrow_, ambient temperature inside the burrow; *M. unguiculatus*, *Meriones unguiculatus*; *M. meridianus*, *Meriones meridianus*.

The distribution of extreme heat in the study area over the day is shown in Figure 7. For *M. unguiculatus*, extreme heat persisted from 11:45 a.m. to 3:45 p.m. at 5 cm from the ground. On the desert surface, the extreme heat lasted from 9:15 a.m. to 6:30 p.m. On the vegetation-covered desert surface, the extreme heat continued from 12:00 a.m. to 4:00 p.m. In the burrow, no extreme heat was measured. In addition, for *M. meridianus*, extreme heat persisted from 9:15 a.m. to 6:30 p.m. at 5 cm from the ground. On the desert surface, the extreme heat lasted from 8:15 a.m. to 7:00 p.m. On the vegetation-covered desert surface, the extreme heat continued from 10:00 a.m. to 6:30 p.m. In the burrow, no extreme heat was measured.

## 4. Discussion

Rodents inhabiting the Mu Us Desert were influenced by extreme heat events during the summer. Ambient temperatures in the near-surface space showed a clear variance within small scales due to the vegetation cover and burrow system. Moreover, high temperatures beyond thermal tolerance limits were detected in the near-surface space frequented by rodents.

### 4.1. Vegetation Coverage and Rodent Species in the Mu Us Desert

Desert vegetation provides rodents with a habitat and the resources they need to survive. Our study showed that the vegetation coverage in the study area is 33.5–40.8%. The vegetation was composed of shrubs dominated by *Salix cheilophila*, *Caragana intermedia*, and *Artemisia ordosica* and a small number of herbaceous plants. Moreover, the vegetation cover in most areas of the Mu Us Desert has increased rapidly in the last 30 years, driven by both climate change and human activities [12,17,35]. Climate change may alter the seasonal temperature and precipitation patterns. Studies have shown an increasing trend in precipitation and temperature in northwest China [36,37,38], which will likely favor desert vegetation restoration. In addition, human activities, such as fencing and vegetation construction, are important forces of desert vegetation rehabilitation [39,40]. In conclusion, future trends in the vegetation coverage of the Mu Us Desert may be favorable for rodent survival.

The Mu Us Desert is a habitat for many species of rodents. In the present study, *M. meridianus*, *M. unguiculatus*, and *C. longicaudatus* were found. However, our data do not provide a full picture of the rodent species in this desert. In fact, *Phodopus roborovskii*, *Cricetulus barabensis*, *Allactaga sibirica*, and *Spermophilus dauricus* also live there [20,41]. In addition, no rodents were caught in simple *Salix cheilophila*-dominated habitats, while 10 rodents were caught in *Caragana intermedia*- and *Artemisia ordosica*-dominated habitats. This may indicate that, together with vegetation coverage, the increased plant species in the habitat also favor the survival of rodents [42]. Moreover, the number of rodents captured in this study was quite low. It is hard to provide effective information on population density. Yet, rodents of all 3 species were captured before 6 am, suggesting that relationships among rodent species may be affected in the face of the combined pressures of climate change and habitat modification [43]. Taken together, although the trends in the vegetation cover of the Mu Us Desert are positive for rodents, changes in the rodent population density and interspecific relationships require further study.

### 4.2. Temperature Variation of Near-Surface Space in Summer

Extreme heat in the summer is becoming more frequent, longer lasting, and hotter. Evidence can be seen from both climate model predictions and weather station detections [6]. Although the temperature data from weather stations differ from the real ambient temperatures experienced by rodents, data from nearby weather stations can be used to further predict the ambient temperatures in rodent habitats. In this study, we found a significant difference between the near-surface ambient temperature and data from the official website (T_air_). The daily maximum T_air_ was 10.2 °C lower than T_5cm_ and 29.6 °C lower than the desert surface temperature (T_0cm_) in July 2022. Moreover, a linear relation between T_air_ and the near-surface ambient temperature was established. These results indicated that the extreme heat experienced by desert rodents in summer was likely underestimated.

In the Mu Us Desert, the vegetation cover and burrow system are effective in regulating the temperature surrounding rodents. In the present study, the vegetation cover reduced the maximum daily desert surface temperature to 65 percent, whereas it had little effect on the air temperature near the desert surface. In addition, the daily maximum temperature in the rodent burrow was 47% of the desert surface temperature. It had a good insulation function at night. However, the study presented measured temperature changes at a depth of 15 cm in the burrow alone. In fact, the structure of rodent burrows is complex. The temperature environment in the burrows is also very diverse. Further research is needed on the role of burrows in rodent summer heat adaptation.

### 4.3. Strategies for Rodent Response to Summer Heat

TNZ is defined as a range of ambient temperature, at which the metabolic rate is at basal or resting levels [44]. This concept provides us with a lot of information on the ability of species to survive in different ambient temperatures. Rodents will be faced with severe physical and physiological limitations when the ambient temperature is above the TNZ. Here, the concept of the TNZ was used to analyze whether rodents experience heat stress during the summer. *M. unguiculatus* exhibits a wide TNZ, and its upper critical temperature is 38 ± 1 °C [21,22,24]. Moreover, this species is able to survive for the long term at 37 °C [23,45]. In contrast, the range of the TNZ in *M. meridianus* is only 38.3% of that in *M. unguiculatus*, and the upper critical temperature is 30.1 °C. In brief, the small desert rodents vary widely in their sensitivity and regulatory limits to variations in ambient temperature [31].

Desert surface temperature, air temperature near the desert surface, and T_burrow_ may reflect the true temperature experienced by small desert rodents. In the study area, the maximum desert surface temperature was 61.8 °C, with a diurnal temperature difference of 46.1 °C. The maximum air temperature near the desert surface was 41.3 °C, with a diurnal temperature difference of 26.2 °C. In contrast, the maximum T_burrow_ was 31 °C, with a diurnal temperature difference of 7.5 °C. Furthermore, outside the burrows, both *M. meridianus* and *M. unguiculatus* were at risk from extreme summer heat. Therefore, summer in the Mu Us Desert is a season of stress and survival for rodents, but burrows may provide them with a place to escape the extreme heat.

## 5. Conclusions

Climate change is changing the pattern of distribution of extreme summer heat in the Mu Us Desert. The evidence suggested that both *M. meridianus* and *M. unguiculatus* living in the southeastern Mu Us Desert were affected by extreme summer heat. This also implies that the behavior, physiology, and interspecies relationships of rodents in this region are expected to change significantly in the near future. Furthermore, burrows can help rodents escape the stress of summer heat, suggesting that, at least in the present study, desert rodents may respond to increased summer heat extremes primarily through behavioral adjustments.

## Figures and Tables

**Table 1:** Plant species, height, and coverage in the Mu Us Desert.

Study Site	Scientific Name	Plant Height (m)	Plant Coverage (%)
Site 1	*Salix cheilophila*	1.904 ± 0.256	40.44
	*Artemisia ordosica*	0.7	0.38
Site 2	*Caragana intermedia*	1.625 ± 0.178	22.17
	*Artemisia ordosica*	0.535 ± 0.176	11.36
	*Cynanchum thesioides*	-	-
Site 3	*Artemisia ordosica*	0.610 ± 0.188	26.40
	*Caragana intermedia*	1.165 ± 0.191	7.85
	*Amygdalus pedunculata Pall.*	0.700 ± 0.100	2.42
	*Cynanchum thesioides*	-	-
	*Psammochloa villosa*	-	-

## Data Availability

The data used in this study are available from the first author upon reasonable request.
